# Testing the competition-colonization trade-off and its correlations with functional trait variations among subtropical tree species

**DOI:** 10.1038/s41598-019-50604-3

**Published:** 2019-10-18

**Authors:** Yue Bin, Guojun Lin, Sabrina E. Russo, Zhongliang Huang, Yong Shen, Honglin Cao, Juyu Lian, Wanhui Ye

**Affiliations:** 1 Key Laboratory of Vegetation Restoration and Management of Degraded Ecosystems, South China Botanical Garden, Chinese Academy of Sciences, Guangzhou, Guangdong 510650 China; 2 Center for Plant Ecology, Core Botanical Gardens, Chinese Academy of Sciences, Guangzhou, Guangdong 510650 China; 3 Changjiang Water Resources Protection Institute, Qintai Road 515, Hanyang District, Wuhan China; 4 School of Biological Sciences, University of Nebraska, Lincoln, NE 68588-0118 USA; 5 Department of Ecology, School of Life Sciences/State Key Laboratory of Biocontrol, Sun Yat-sen University, Guangzhou, 510275 China

**Keywords:** Forest ecology, Biodiversity

## Abstract

The competition-colonization trade-off, by which species can partition spatial niches, is a potentially important mechanism allowing the maintenance of species diversity in plant communities. We examined whether there was evidence for this trade-off among tree species in a subtropical forest and how it correlated with eight functional traits. We developed and estimated a metric for colonization ability that incorporates both fecundity and seed dispersal based on seed trap data and the sizes and distributions of adult trees. Competitive ability was estimated as survival probability under high crowding conditions based on neighborhood models. Although we found no significant relationship between colonization and competitive abilities, there was a significant negative correlation between long distance dispersal ability and competitive ability at the 5 cm size class. Colonizers had traits associated with faster growth, such as large leaves and low leaf lamina density, whereas competitors had traits associated with higher survival, such as dense wood. Our results imply that any trade-off between competition and colonization may be more determined by dispersal ability than by fecundity, suggesting that seed dispersal is an important contributor to diversity maintenance. Future work should test how competitive ability covaries with the components of colonization ability, as we did here.

## Introduction

Understanding the mechanisms responsible for species coexistence, particularly in species-rich plant communities, is important if we are to predict how communities will respond to anthropogenic influences, such as forest fragmentation and climate change^1^. One important mechanism involves the partitioning of spatial niches via a trade-off between species’ abilities to colonize space with propagules versus to displace other species, *i*.*e*., long-run competitive dominance^2^, such that species that are inferior competitors are better colonizers, and vice versa^3–5^. In such spatially structured communities, competitively inferior species can occupy vacant sites and establish populations before competitively superior species arrive and eventually displace them. In early theoretical models of such competition-colonization trade-offs^3^, competitive ability was defined as the immediate displacement of a competitively inferior species, and colonization ability was defined as the ability to arrive at a recruitment site. Later models used the more realistic assumption of replacement competition, in which colonizers of a site compete with each other as juveniles to win the site, assuming that the adult dies^5,6^. In such models, purely spatial subdivision is insufficient for species to stably coexist, and some form of environmental heterogeneity is required, along with appropriate trade-offs involving colonization^6^.The necessary environmental heterogeneity can take a variety of forms, including variation in resource-limitation stress^1^, the density of patches suitable for recruitment^6^, or successional niches^5^. It is not clear which of these underlying processes predominantly operates in natural plant communities to contribute to diversity maintenance, and it is likely that several operate simultaneously. However, an important step in evaluating the importance of such trade-offs in natural plant communities is to test whether there is covariation among species in colonization ability and competitive ability.

Competitive ability is often defined in different ways^7–11^, but in the context of the colonization-competition trade-off, it is defined as the ability of a species to survive and displace another individual of a different species at a particular site, given that at least one seed of that species arrived there^6^. Following this definition, many studies have considered species that did not survive as weaker competitors than the species that survived and displaced them^8,9^.

While plants compete for many types of resources, in forests, a key limiting resource is light^12^, with disturbances creating canopy gaps in otherwise shaded conditions. In forests ranging from the temperate zone to the tropics, coexisting tree species have been observed to vary widely in the ability to colonize potential recruitment sites, as determined by fecundity and dispersal^13–15^. Similarly, there is also substantial variation among tree species in the ability to survive in shaded environments, which in forests, are areas of high neighborhood crowding by trees^12,16^. Since in the colonization-competition trade-off, a species’ competitive ability is defined as its ability to survive and displace other species, the ability to survive at high neighborhood crowding is a good measure of competitive ability, because in the long-run such species will win competition with species that cannot survive crowding. However, only a few studies have examined covariation between colonization ability and survival as a way to explore the importance of the competition-colonization trade-off in forest communities^12,14,17^.

A species’ colonization and competitive abilities are determined in part by its functional properties. Colonization ability is a function of both fecundity, which often varies inversely with seed size within plant growth forms (the stature of the species at maturity^18^), as well as seed dispersal, which determines how many sites can be reached by the seeds an individual produces^14,19^. While plant competition has been viewed from several perspectives^2,20^ competitive ability in forests is often considered to correlate with species survival rate in shade^12^. Larger-seeded species are generally more shade tolerant as seedlings and are thus considered better competitors since they can outlive species with greater demands for light^21,22^. Smaller-seeded species, in contrast, are considered better colonizers^7^, since they are often more fecund and can be dispersed by a wider array of agents^23^. Although larger-seeded animal-dispersed species can be well-dispersed^19^, they may not always be better colonizers, since larger seed size is often associated with reduced fecundity^1,18^.

Many other functional traits covary with seed size and fall along well-established axes of ecological strategy space that contrast fast-growing, light-demanding species having low survival with slower-growing, shade-tolerant species having high survival. Tissue density contributes to physical strength, durability, and longevity^24^, and so species with high wood^25^ and leaf lamina densities^26,27^, low specific leaf area (SLA)^26^, and high leaf dry matter content (LDMC)^26^ tend to tolerate shade better and thus have higher survival rates. In contrast, faster-growing, light-demanding species tend to be lighter-wooded, with high SLA, low LDMC, and other traits that allow the inherently fast growth rates that are associated with low species survival rates^26,28^.Thus, the competition-colonization trade-off should align with trait variation associated with interspecific variation related to dispersal, growth, survival and shade tolerance.

While the competition-colonization trade-off is viewed as a species property, these abilities also vary through ontogeny and with growth form. As a tree grows, its access to exogenous resources changes, as does its allocation of endogenous resources to functions such as growth, survival, and reproduction, all of which would influence the trade-off axis^29^. Likewise, tree species differ in asymptotic height, which strongly affects access to and allocation of resources affecting these vital rates^30,31^. It is therefore important to account for variation in this trade-off with respect to ontogeny and growth form, as multispecies coexistence in forests depends upon the abilities for tree populations to be maintained and progress through different size classes to maturity.

Here, we assess whether there is any evidence for a competition-colonization trade-off among 13 tree species in a 20- ha fully mapped, long-termed monitoring plot in subtropical forest in southern China and examine whether functional traits covary with the competition-colonization relationship. We used inverse models parameterized with ten years of seed-rain data to estimate the seed dispersal curve and size-specific fecundity for each species, which we then used to estimate the time for a species to colonize a vacant site as an inverse measure of colonization ability for three tree sizes. Since fecundity and dispersal are two components of colonization ability, we also estimated long-distance dispersal ability based on the seed dispersal curve. Competitive ability was estimated at three tree sizes for each species as survival probability under conditions of high neighborhood crowding^12,32^, which is negatively related with light availability^32,33^. Based on these measures, we tested whether there was any evidence for a competition-colonization trade-off among species in this forest, which we evaluated based on whether there was a significant negative correlation between species’ competitive and colonization abilities. We also examined the relationship of the two components of colonization ability (fecundity and long distance dispersal ability) with competitive ability, to evaluate whether a competition-colonization trade-off might be driven more by fecundity or dispersal. While a significant correlation may arise even if there is no true trade-off^34^, if a trade-off is present, we reason that there should be evidence for it in the form of a negative relationship between these abilities.

Investigating the functional basis of the competition-colonization trade-off, if present, can improve our understanding of the physiological underpinnings of plant strategies relevant for species coexistence. To evaluate whether any trade-offs observed may have a functional basis, we also investigated whether species’ competitive and colonization abilities were related to variation in functional traits (wood density, seed mass, SLA, LDMC, leaf area, leaf lamina thickness, leaf lamina density, and folia chlorophyll concentration) These commonly used functional traits are considered to correlate with ecological and physiological processes underlying species’ competitive and colonization abilities^7,18,23–28^.

## Results

### Is there evidence for a competition-colonization trade-off?

**Figure 1:**
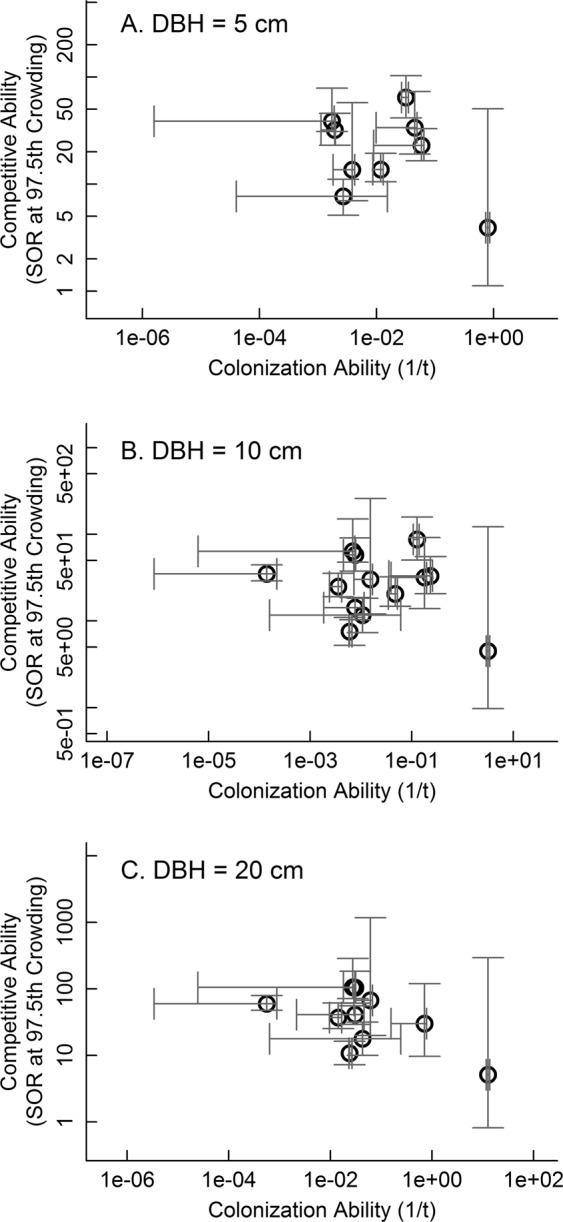
The relationship between colonization and competition abilities for 13 tree species in a subtropical Chinese forest for trees with diameter and breast height (DBH) of 5 (**A**), 10 (**B**), and 20 (**C**) cm. Colonization ability was expressed as the inverse of the time (*t*) required to colonize a gap, so larger values of 1/*t* imply better colonization ability. Competitive ability was expressed as the species’ survival odds ratio (SOR) at the 97.5^th^ percentile of crowding. SOR was calculated as (survival probability)/(1-survival probability). Larger values of SOR imply better competitive ability. The grey segments represent the 95% confidence intervals.

**Figure 2:**
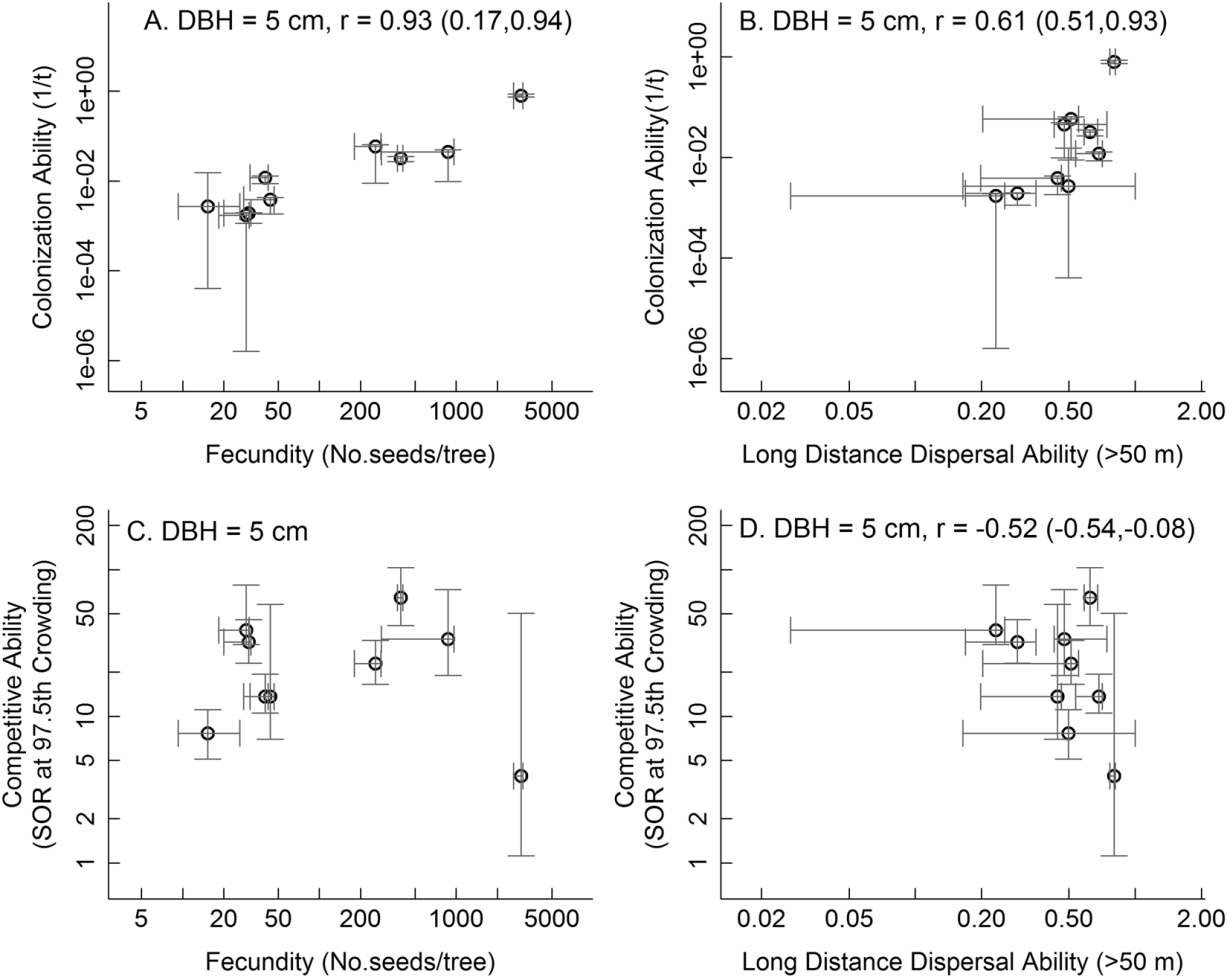
The relationships between colonization ability and its two components, fecundity (**A**) and dispersal (**B**), and between competitive ability and the two components of colonization ability, fecundity (**C**) and dispersal (**D**), modeled for trees with diameter at breast height of 5 cm for 13 tree species representing three growth forms in a subtropical Chinese forest. Figures for other tree diameters are in the appendix (Fig. S1). Correlation coefficients and their probabilities based on all species are reported only for statistically significant relationships. Bootstrapped confidence intervals are given in parentheses beside the correlation coefficients. Colonization ability was expressed as the inverse of the time (*t*) required to colonize a gap, so larger values of 1/*t* imply better colonization ability. Competitive ability was expressed as the species’ survival odds ratio (SOR) at the 97.5^th^ percentile of crowding. SOR was calculated as (survival probability)/(1-survival probability). Larger values of SOR imply better competitive ability. The grey segments represent the 95% confidence intervals.

Among the 13 tree species in our study, we did not find any significant negative relationships between colonization and competitive abilities for the three tree sizes tested. Although the correlation coefficients based on mean estimates of colonization and competitive abilities of the species were negative for all tree sizes, the bootstrapped confidence intervals on the correlation coefficients always included zero (Fig. 1). Across all size classes, colonization ability was positively correlated with both of the two components comprising it, fecundity and long distance dispersal ability (5 cm: Fig. 2A,B; 10 and 20 cm: Fig. S1A,B,E,F), consistent with expectations for a reasonable measure of colonization ability. Fecundity and long-distance dispersal ability were, however, differently associated with competitive ability (5 cm: Fig. 2C,D; 10 and 20 cm: Fig. S1C,D,G,H). Competitive ability and fecundity did not show a clear relationship for trees of any size (5 cm: Fig. 2C; 10 and 20 cm Fig. S1C,G). In contrast, our estimate of long distance dispersal ability was negatively correlated with competitive ability, but this relationship was only significant at the 5 cm size (5 cm: Fig. 2D; 10 and 20 cm: Fig. S1D,H).

### Which functional traits are associated with variation in colonization and competitive abilities?

**Figure 3:**
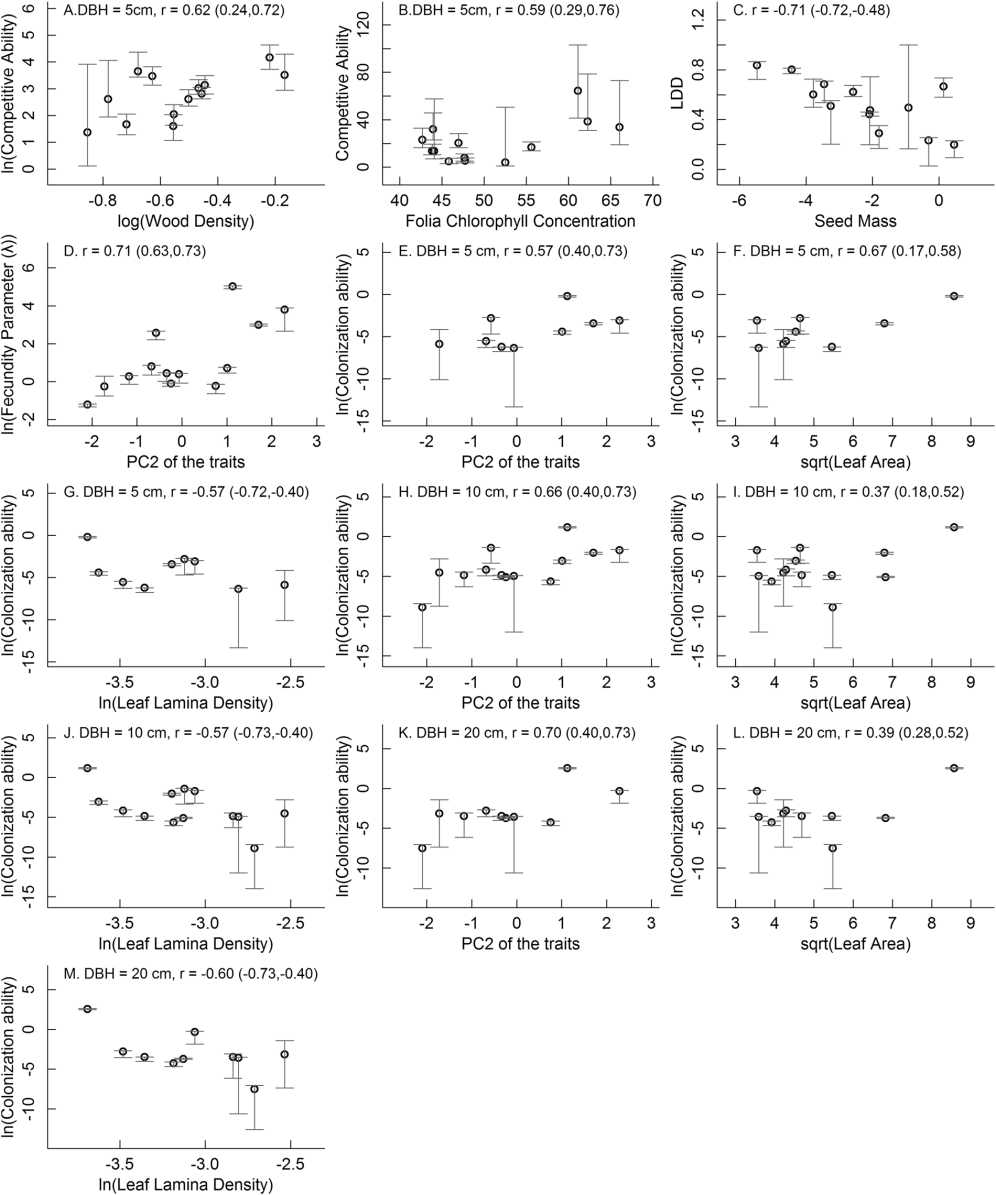
The significant relationships of functional traits, the PCs of the functional traits with competitive ability, colonization ability and the components of colonization ability (fecundity parameter and long-distance dispersal ability) for all 13 species: the correlations of competitive ability at diameter at breast height (DBH) of 5 cm with wood density (**A**) and folia chlorophyll concentration (**B**), of long distance dispersal ability with seed mass (**C**), of the size-independent fecundity parameter with PC2 of the traits (**D**), of PC2 of the traits (**E**,**H**,**K**), leaf area (**F**,**I**,**L**) and leaf laminadensity (**G,J,M**) with colonization ability at DBH of 5 cm (**E**–**G**), 10 cm (**H**–**J**) and 20 cm (**K**–**M**). Bootstrapped confidence intervals are given in parentheses beside the correlation coefficients. Competitive ability was expressed as the species’ survival odds ratio (SOR) at the 97.5^th^ percentile of crowding. SOR was calculated as (survival probability)/(1-survival probability), so larger values of SOR imply stronger competitive ability. The grey segments represent the 95% confidence intervals.

Among the functional traits examined, only wood density and folia chlorophyll concentration had significantly positive correlations with competitive ability at the 5 cm size (Fig. 3A,B). Seed mass was negatively correlated with long distance dispersal ability (Fig. 3C). A positive correlation was found between trait PC2 and the fecundity parameter, which estimates allocation to reproduction per unit basal area, and so, is size-independent (Fig. 3D). Trait PC2 was relatively strongly loaded by leaf lamina thickness and folia chlorophyll concentration (Fig. S2). Colonization ability was significantly positive correlated with trait PC2 and leaf area but negatively correlated with leaf lamina density at all sizes examined (Fig. 3E–K). The scatter plots for all the relationships between functional traits, the PCs of the functional traits and the colonization and competitive abilities are in Figs S3–S10.

## Discussion

The abilities of tree species to compete effectively and to colonize potential recruitment sites have been thought to trade-off with each other^3,4^, since the functional trait values that allow species to be good competitors (*e.g*., large seed size and leaf and wood traits enabling high survival rates) are considered to be incompatible with those that make species good colonizers (*e.g*., small seed size and leaf and wood traits enabling fast growth). While we did find colonization ability to be associated with traits allowing fast growth (large leaf area and lower lamina density^26–28^) and competitive ability to be associated with traits allowing higher survival (high wood density^25^), we found no significant relationship between the competitive and colonization abilities among 13 species in this Chinese subtropical forest. Colonization ability is, however, determined by both fecundity and dispersal distance^14,17^, and there was a significantly negative correlation between long distance dispersal and competitive ability for trees in the 5 cm size class. Our results therefore suggest that any trade-off between competition and colonization may be more determined by dispersal ability than by fecundity and that dispersal may more strongly affect how species partition spatial niches, the mechanism by which the trade-off facilitates species coexistence. Recent studies showing that fecundity displays approximately orthogonal variation to the growth-survival trade-off are also consistent with our finding that fecundity did not directly trade-off with competitive ability in this forest^35^. Future empirical studies of the colonization-competition trade-off should therefore separately quantify and evaluate the fecundity and dispersal components of colonization ability, as we did here, and theoretical models can separately evaluate the ability of fecundity-competition and dispersal-competition trade-offs to allow species to coexist.

Competitive ability measured in our study is largely a function of shade tolerance. Species that are not shade tolerant can only recruit in gaps, and the ability to disperse long distances provides higher probability of finding a gap^24^, so given that a species has low competitive ability, there should be selection for long-distance dispersal, as we found. From a sampling perspective, fecundity influences the probability of realizing long-distance dispersal, given a potential seed shadow determined by dispersal traits. Among our studied species, long-distance dispersal ability was negatively associated with seed mass, consistent with studies showing large, wind-dispersed seeds to be dispersed shorter distances^36^.

Only a few studies have considered the effects of both fecundity and dispersal on colonization ability. In a subtropical forest in Puerto Rico, trade-offs with competitive ability were found for both long distance dispersal ability and fecundity among ten species^12^. Jakobsson and Eriksson found a trade-off among 15 wind-dispersed forb species between competitive ability and dispersal ability measured as a function of terminal velocity^17^. However, when colonization ability was estimated as an index incorporating dispersal ability and fecundity, it did not trade-off with competitive ability among these species, consistent with our findings.

Several other studies have examined the existence of a competition-colonization trade-off, principally among herbaceous species^8–11,37^. Adult longevity^10^, local dominance^11^, the abilities to survive and displace other species at low soil moisture concentration^8^ and at low soil nitrogen concentration^9^, and the ability to displace other individuals at establishment sites^7^ have been correlated with different proxies or components of colonization ability, typically focused on either dispersal ability or fecundity alone. These metrics included dispersal mode^10^, terminal velocity^11,17^, dispersal distance^8^, and fecundity^9^. Without explicitly considering the uncertainty in parameter estimates, as we did here, some of these studies have found a significant negative correlation between competition and colonization^7,9^, while others have not^10,11,37–39^. That these metrics focused on different aspects of competitive and colonization abilities may in part account for their conflicting results.

In our study, both the competitive and the colonization abilities of these species were estimated from models fitted to extensive data on tree survival and seed dispersal, and thus encompass individual variation among tree during these life stages. However, without explicitly accounting for the sources of individual variation, for example, the environment of each tree, substantial variation exists around the mean estimates of competitive and colonization abilities, as observed for other demographic rates^40^. Moreover, seed dispersal and mortality can be affected by stochastic processes, leading to further uncertainty in the estimation of colonization and competitive abilities. Such unaccounted sources of variation can obscure the ability to detect any underlying colonization-competition trade-off, if one was present. Calibrating these models based on controlled experiments or long term observations that cover the entire life history of these tree species may provide better fits of the fecundity, dispersal and survival models and thus better estimates of colonization and competitive abilities.

The few significant trait relationships with competitive and colonization abilities in our study are in keeping with a growing body of work showing that interspecific functional variation and demographic rates are often not strongly related to each other^41,42^. Our results suggest that a strongly deterministic functional basis for a trade-off between colonization-competition is unlikely, which may explain why robust evidence for it has not consistently been observed. We focused on interspecific variation in functional traits and colonization and competitive abilities, and if there was substantial intraspecific variation, it could have obscured our ability to detect how traits determine this trade-off^43^. Moreover, plant functional traits are complex, and variation in them is also shaped by phenotypic integration^44^. A single functional trait can thus be related to multiple functions, which may further act to obscure our ability to detect significant trait relationships with colonization or competitive abilities, which are themselves also complex traits.

## Conclusion

Although theoretical studies have suggested the importance of competition-colonization trade-off to species coexistence^2–4^, our study showed little evidence for it when fecundity is incorporated into the estimate of colonization ability. Instead, we found that what trades off more strongly with competitive ability is dispersal ability, suggesting the existence of a competition-dispersal trade-off. Thus, the partitioning of spatial niches, which allows for the colonization-competition trade-off to facilitate coexistence, may be mediated more by dispersal than fecundity.

## Methods

### Study site and tree species data

Our study site was in Dinghushan Nature Reserve (DHS) (112°30′39″–112°33′41″E, 23°09′21″–23°11′30″N) in Southern China. This region is characterized by a south-subtropical monsoon climate, with a mean annual temperature of 20.9 °C and mean annual precipitation of 1929 mm. A 20-ha plot was established in 2005 in the subtropical evergreen broadleaved monsoon forest in the nature reserve. All stems with diameter at breast height (DBH) ≥ 1 cm were mapped, tagged, measured, and identified to species. The plot was re-censused in 2010. In the 2005 census, there were 71617 individuals, falling into 210 species (all with evergreen leaf habit), 119 genera, and 56 families, the total basal area of the plot was 282365 cm^2^, and overall forest canopy height was about 30 m. Fagaceae, Theaceae, Juglandaceae and Lauraceae are the dominant families in the plot^45^.

Seeds in the DHS plot have been collected twice a month since November 2008 from 149 seed traps (traps are 0.5 m^2^ in area), arranged along the trails in the plot (Fig. S11). For this study, seed rain data over ten years were available (2008–2018), comprising 35 species, of which 13 had sufficient sample sizes (at least 250 seeds collected and present in a minimum of 20 traps) for estimation of colonization ability (Table 1). Our study species encompassed three growth forms, including three understory, three midstory and seven canopy species.

**Table 1:** Information on the taxonomy, growth form (canopy, midstory, understory), shade tolerance (tolerant, medium, intolerant), number of stems, total basal area (cm^2^), diameter at breast height (DBH, measured in cm) representing the reproductive size threshold (DBH_*r*_) and maximum observed size (DBH_*m*_) of each focal species, with abbreviation codes for the species’ scientific name.

Latin binomial	Species Code	Family	Growth Form	Shade Tolerance	No. of Stems	Total Basal Area	DBH_*r*_	DBH_*m*_
*Mallotus paniculatus*	MP	Euphorbiaceae	Midstory	Intolerant	146	210.7	2.5	23.8
*Memecylon ligustrifolium*	ML	Melastomataceae	Midstory	Tolerant	1263	880.4	3.0	33.3
*Ormosia glaberrima*	OG	Leguminosae	Canopy	Medium	2702	2842.0	4.0	36.5
*Aidia canthioides*	AC	Rubiaceae	Understory	Tolerant	5996	1998.6	1.5	17.2
*Schima superba*	SS	Theaceae	Canopy	Medium	2296	38668.5	6.0	89.0
*Cryptocarya chinensis*	CC	Lauraceae	Canopy	Medium	2557	11239.0	5.0	48.0
*Machilus chinensis*	MC	Lauraceae	Canopy	Medium	532	8250.3	5.0	63.0
*Engelhardtia roxburghiana*	ER	Juglandaceae	Canopy	Intolerant	737	31215.5	8.0	95.0
*Ardisia quinquegona*	AQ	Primulaceae	Understory	Medium	3702	690.4	1.0	17.0
*Acmena acuminatissima*	AA	Myrtaceae	Canopy	Tolerant	1484	10265.1	6.0	63.0
*Artocarpus styracifolius*	AS	Moraceae	Midstory	Medium	388	1900.4	3.0	35.1
*Aporosa yunnanensis*	AY	Phyllanthaceae	Understory	Tolerant	3747	4184.6	2.5	17.0
*Castanopsis chinensis*	Cc	Fagaceae	Canopy	Medium	2311	86580.0	6.0	175.0

In 2012, six functional traits (wood density, SLA, LDMC, leaf area, folia chlorophyll concentration and leaf lamina thickness) for the species in the DHS plot have been measured from leaf and wood samples collected for each species, using the standardized methods of Cornelissen *et al*.^46,47^. With SLA and leaf lamina thickness, we calculated leaf lamina density as 1/(SLA × thickness). The average trait value across samples for a species was used in analyses. The datasets generated during and/or analyzed during this study are available from the corresponding author on reasonable request.

### Modeling colonization ability

Some consider colonization to include the seedling establishment stage^17^, but this confounds processes related to the ability to arrive at a site versus the ability to establish there, and the latter is related to competitive ability in forests. We therefore consider colonization to be seed arrival to a site, consistent with theoretical studies^3,4^. Fecundity and dispersal are the two main determinants of colonization ability, and we used inverse modeling and data on seed rain into seed traps, the sizes of reproductive trees, and locations of trees and seed traps to estimate them simultaneously based on likelihood functions incorporating fecundity and alternative dispersal kernels.

For the fecundity function, we defined a fecundity parameter that estimates allocation to reproduction per unit basal area, and so, is size-independent. We assumed that size-specific fecundity was a linear function of the size-independent fecundity parameter and the basal area of a reproductive tree, following many previous studies^13,48^. Species-specific reproductive size thresholds were obtained from experts working in the DHS (Huang, Z. & Cao, H., pers. comm; Table 1). To model the probability of seed arrival as a function of distance from a mother tree, we evaluated support for four dispersal kernels widely used for estimating seed dispersal curves: the negative exponential, two dimensional *t* (2Dt), lognormal, and Weibull probability distribution functions^14,15,19^. With fecundity and dispersal kernels, we calculated the expected seed number to a seed trap for each conspecific adult tree. We then summed up the contribution of each adult tree and obtained the expected number of seeds falling into each seed trap. We assumed a Poisson distribution for the distribution of the observed seed number given the expected seed number for a seed trap. By maximizing the likelihood function, we simultaneously found the best estimates for the parameters of the fecundity function (the fecundity parameter) and of each dispersal kernel (*b*_1_ and *b*_2_ in Table A1 in Appendix 1). Details were given in Appendix 1 of the supplementary file. According to the Akaike Information Criterion (AIC), the dispersal kernel with the lowest AIC for each species was selected for subsequent analysis^49^.We calculated the probability of long-distance (>50 m) dispersal (size-independent) as $LDD=1-{\int }_{0}^{50}2\pi rP(r)dr$, where *P*(*r*) is the best-fitting dispersal kernel for each species.

With the size-specific fecundity and best dispersal kernel for each species, we estimated a size-specific colonization rate as the inverse of the time needed for a single mother tree at the center of the plot to colonize a 10 × 10 m gap randomly located in the plot. This gap size was chosen because it approximated the crown projection area of a typical canopy tree in DHS. We calculated the probability for a seed to arrive at the gap, *p*_G_, by integrating the dispersal kernel over the gap area using the *cubature* R package^50^. Even though there is only one mother tree, integration over the gap area (space) is needed in order to obtain the probability for a seed to land in the gap, since we need the cumulative probability of seed arrival for the part of the kernel covering the gap area.

The arrival of a seed can be treated as an independent Bernoulli trial. The probability that a seed is the first to arrive in the gap follows a geometric distribution, and the expected number of seeds required for the first arrival is 1/*p*_G_. Hence, the expected number of years (*t*) for first arrival is $t=1/({p}_{{\rm{G}}}F)$, where *F* is fecundity. We used 1/*t* as a measure of a species’ colonization ability. Because fecundity is a function of diameter, 1/*t* is also influenced by tree size, and we calculated 1/*t* for trees of 5, 10, and 20 cm in diameter. We used a single mother tree here so that colonization ability is as an inherent property of a species determined only by tree size, fecundity, and dispersal properties, rather than by the abundance of reproductive trees in the plot, which can vary through time.

The confidence intervals for the colonization ability and the parameters of the seed dispersal models were obtained by bootstrapping. For each of the 1000 simulations, the same number of seed traps as observed were randomly selected from our full data with replacement, and the parameters were estimated for those samples, yielding 1000 estimates for every parameter of the seed dispersal models, as well as the colonization ability. The 2.5^th^ and the 97.5^th^ percentiles of the distributions of these estimates were used as their 95% confidence intervals.

### Modeling competitive ability

Following Uriarte *et al*.^12^, competitive ability of a species was estimated as its average survival probability in high crowding conditions. For each species, we used logistic regression to fit survival probability of each tree, with a logit link function, as a linear function of its initial diameter and its neighborhood crowding (NC) index. Following Comita *et al*.^51^, NC was calculated as

1 $$N{C}_{i}=\,\mathrm{ln}(\mathop{\sum }\limits_{j=1}^{nneighbors}(\pi {({D}_{j}/2)}^{2}\exp (\,-\,0.2{d}_{ij})))$$

where *nneighbors* is the total number of neighbors with larger diameters, and within 15 m of the focal tree, *D*_j_ is the diameter of the *j*^th^ neighbor, *d*_ij_ is the distance of *j*^th^ neighbor to the *i*^th^ tree^49^. We assumed that focal trees were shaded only by taller stems, and, although we lack species-specific height- diameter allometries, taller stems usually have larger diameters. We limited model fitting to focal trees >15 m from the plot’s edge to ensure complete neighborhood information.

Size-specific competitive ability was estimated from these species-specific fits as the predicted survival probability at the 97.5^th^ percentile of neighborhood crowding (NC) for all individuals across the plot and for trees with diameters of 5, 10, and 20 cm. Survival probability of a species at a given diameter was not predicted if that diameter was larger than the observed maximum diameter for that species. Survival probability (*s*) (varying from zero to one) was presented as the survival odds ratio (SOR), which is *s*/(1-*s*) and varies from zero to positive infinity. The confidence intervals for parameters of the survival models were given by1000 resampling over 20 × 20 m quadrates. Each time, we randomly sampled with replacement the same number of quadrats as the species occupied. Then the survival model was fit for this random sample 1000 times, yielding 1000 estimates for every parameter of the survival model. The 2.5th and 97.5th pencentiles of the distribution of a parameter was used as its 95% confidence interval. We sampled over the quadrats rather than on the individual so that the spatial correlation of survival probability among the individuals could be maintained.

Random samples were drawn from each species’ distributions of the bootstrapped estimates of colonization and competitive ability for 1000 times. For each draw, the estimates were correlated across species, producing a distribution of correlation coefficients. The 2.5^th^ and 97.5^th^ percentiles of this distribution were used as the confidence interval for the correlation coefficient. A similar procedure was used to estimate the confidence intervals for the correlations of competitive and colonization abilities with long distance dispersal ability and the fecundity parameter.

### Colonization ability and competitive ability in relationship to functional traits

We considered a statistically significant negative correlation between 1/*t* and SOR under high crowding to be evidence of a competition-colonization trade-off. To test the correlations between colonization ability, competitive ability and functional traits, principle components analysis (PCA) was performed on functional trait data. The first three trait PCs explained 78.9% of the total variance in traits. The trait PC1 was positively related to leaf area and SLA and negatively correlated with the other functional traits (Fig. S2), suggesting that larger scores of PC1 were consistent with trait variation commonly seen in more light-demanding species. Leaf lamina thickness and folia chlorophyll concentration had strong loadings of 0.576 and 0.495 on trait PC2. Leaf area and LDMC had the strongest loading of 0.609 and 0.606 on trait PC3. For the significant trait correlations with abilities, bootstrap samples of competitive ability and components of colonization ability were also correlated with the trait values for confidence intervals.

## Supplementary information

Appendix

## Data Availability

Materials and correspondence should be addressed to Wanhui Ye, why@scbg.ac.cn.
