# Supplementary material for: Testing the competition-colonization trade-off and its correlations with functional trait variations among subtropical tree species
Source: Sci Rep. 2019 Oct 18;9:14942. doi: 10.1038/s41598-019-50604-3 (PMC6802185; doi:10.1038/s41598-019-50604-3)
Supplement: Supplementary file 1 — Appendix [file 41598_2019_50604_MOESM1_ESM.docx]

**Electronic Supplementary Material**

Testing the competition-colonization trade-off and its correlations with functional trait variations among subtropical tree species

Yue Bin ^1,2^_,_Guojun Lin^3^, Sabrina E. Russo^4^, Zhongliang Huang ^1,2^, Yong Shen^5^, Honglin Cao^1,2^, Juyu Lian^1,2^, Wanhui Ye^1,2*^

^1^Key Laboratory of Vegetation Restoration and Management of Degraded Ecosystems, South China Botanical Garden, Chinese Academy of Sciences, Guangzhou 510650, China.

^2^Guangdong Provincial Key Laboratory of Applied Botany, South China Botanical Garden, Chinese Academy of Sciences, Guangzhou 510650, China

^3^Changjiang Water Resources Protection Institute, Qintai Road 515, Hanyang District, Wuhan, China.

^4^School of Biological Sciences, University of Nebraska, Lincoln, NE, USA 68588-0118; srusso2@unl.edu

^5^Department of Ecology, School of Life Sciences/State Key Laboratory of Biocontrol, Sun Yat-sen University, Guangzhou 510275, China

*Author for correspondence

E-mail: why@scbg.ac.cn

Fax Number: 86-20-37252981

Figure S1.


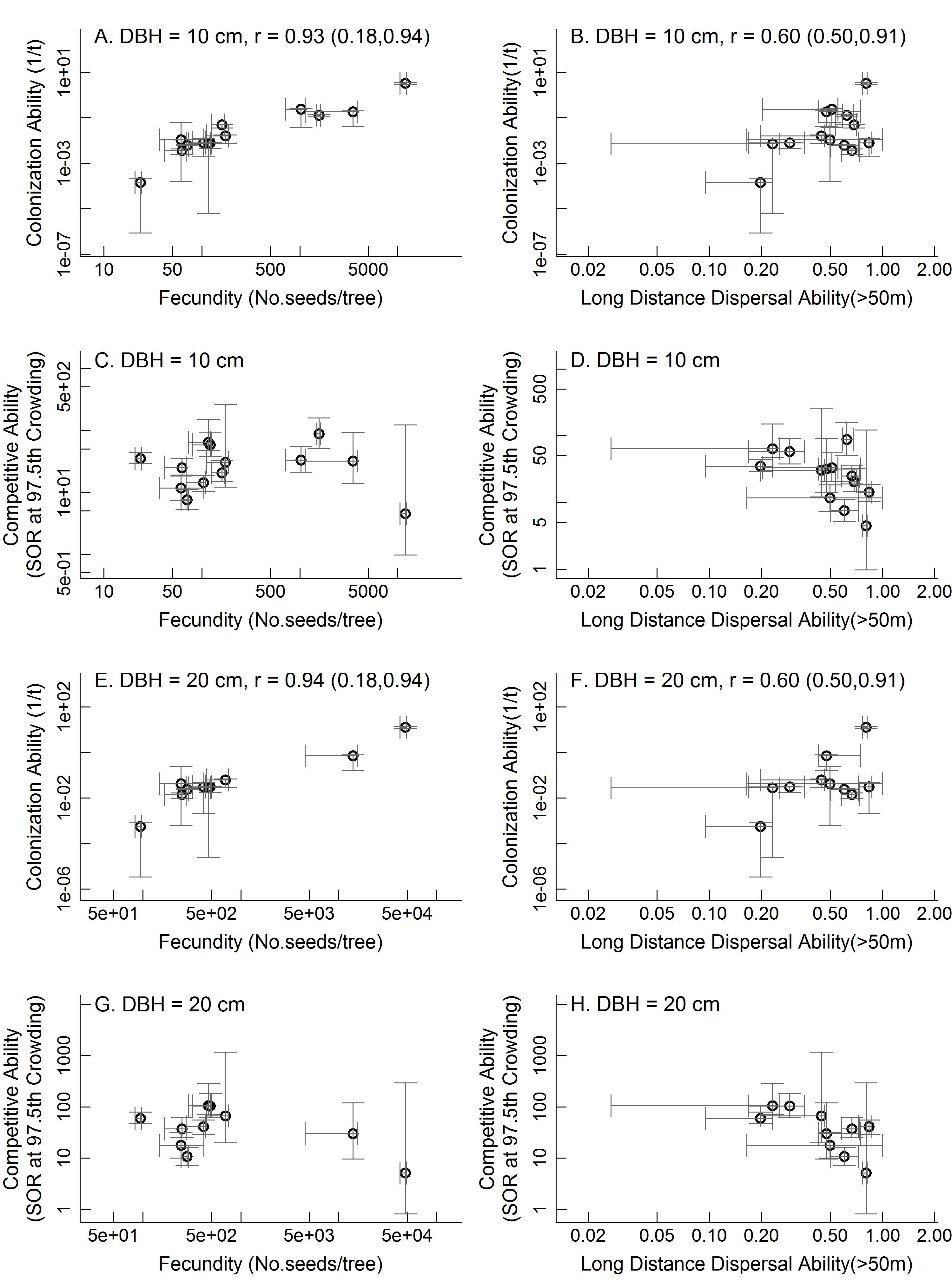


**Fig. S1.** The relationships between colonization ability (the inverse of colonization time) and its two components, fecundity (A, E) and dispersal (B, F), and between competitive ability and the two components of colonization ability, fecundity (C, G) and dispersal (D, H), modeled for trees with diameter at breast height of 10 and 20 cm for 13 tree species. Correlation coefficients and their probabilities were reported only for statistically significant relationships. Bootstrapped confidence intervals were given in parentheses beside the correlation coefficients. See Figure 2 for the relationships for trees with diameter of 5 cm. Colonization ability was expressed as the inverse of the time (*t*) required to colonize a gap, so larger values of 1/*t* imply better colonization ability. Competitive ability was expressed as the species’ survival odds ratio (SOR) at the 97.5^th^ percentile of crowding. SOR was calculated as (survival probability)/(1-survival probability). Larger values of SOR imply better competitive ability.

Figure S2.


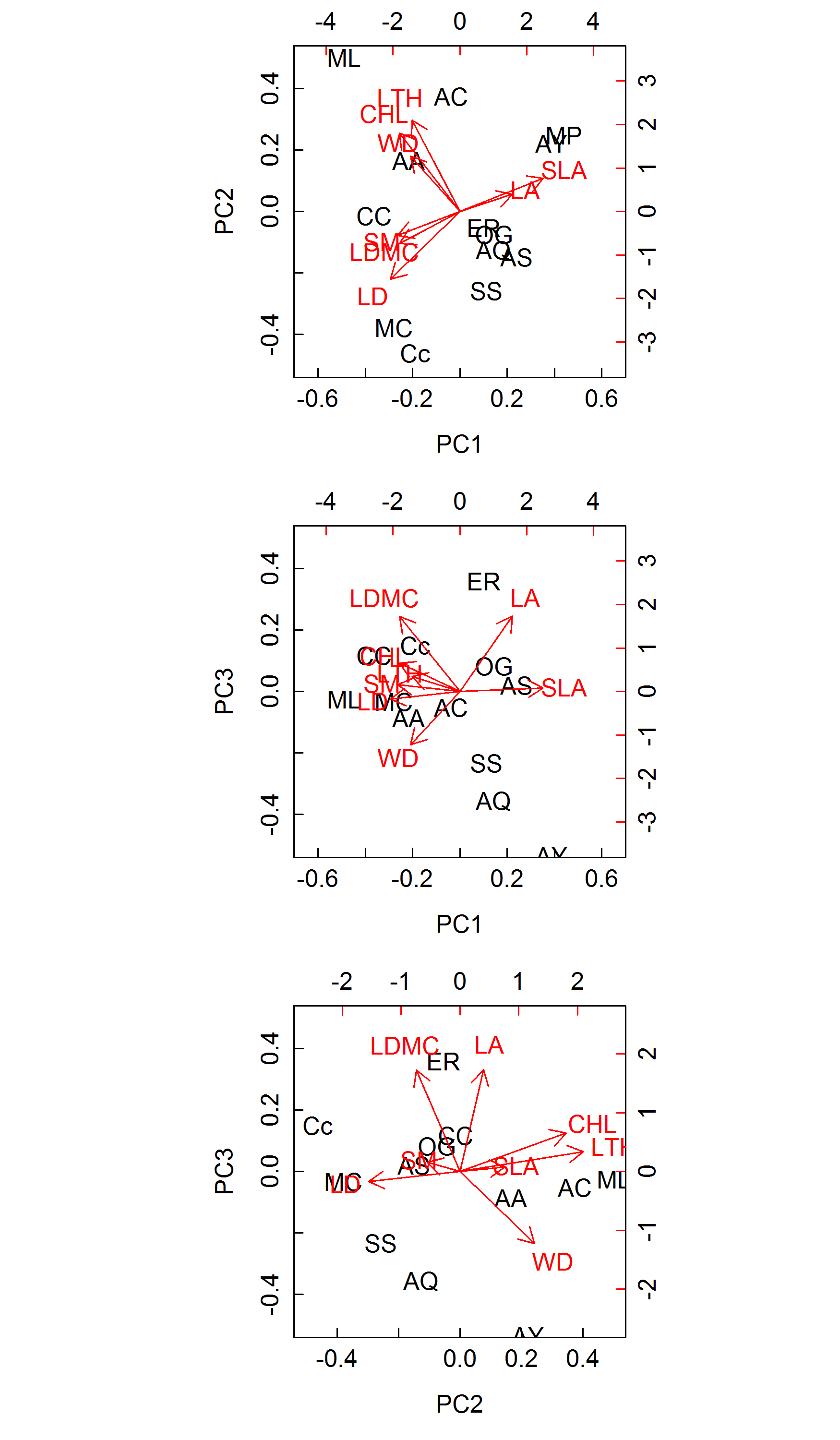


**Fig. S2.** Biplots of the principle components of functional traits of 13 tree species in a subtropical forest in China. Black and red letter combinations denoted species and traits, respectively. MP: *Mallotus paniculatus*; ML: *Memecylon ligustrifolium*; OG: *Ormosia glaberrima*; AC: *Aidia canthioides*; SS: *Schima superba*; CC: *Cryptocarya chinensis*; MC: *Machilus chinensis*; ER: *Engelhardtia roxburghiana*; AQ: *Ardisia quinquegona*; AA: *Acmena acuminatissima*; AS: *Artocarpus styracifolius*; AY: *Aporosa yunnanensis*; Cc: *Castanopsis chinensis*; CHL: folia chlorophyll cencentration; LA: leaf area; LTH: leaf lamina thickness; LDMC: leaf dry matter content; WD: wood density; SLA: specific leaf area; SM: seed mass; LD: leaf lamina density.

Figure S3.


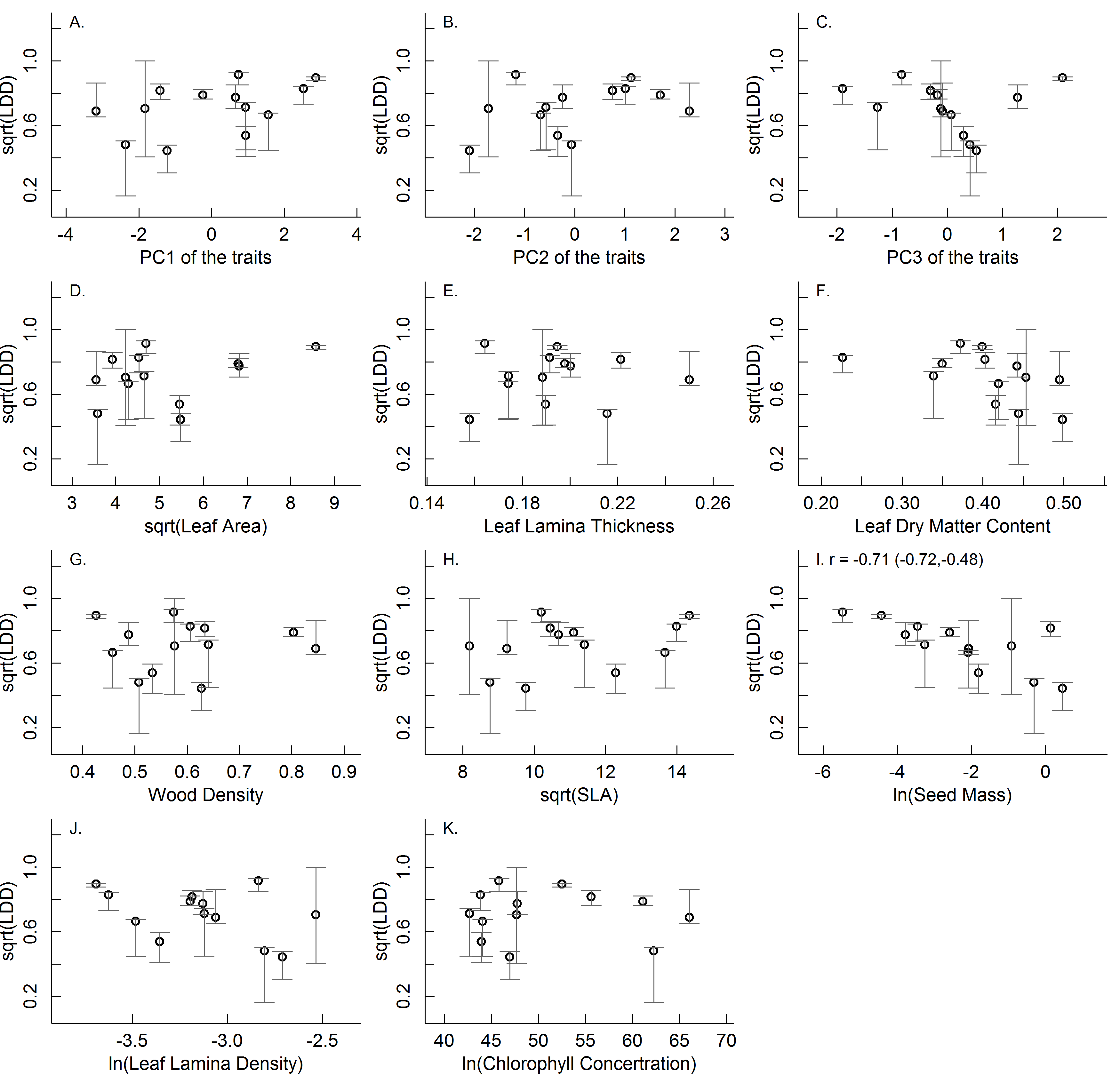


**Fig. S3.** The relationships of long distance dispersal ability with the first to the third principal components (PC1, PC2, PC3) of the functional traits (A, B, C) and each functional trait (D to K) for tree species in a 20-ha subtropical forest dynamic plot in China. The functional traits included leaf area (D), leaf lamina thickness (E), leaf dry matter content (F), wood density (G), specific leaf area (SLA; H), seed mass (I), leaf lamina density (J) and folia chlorophyll concentration (K) . Long distance dispersal ability was square-root transformed. Leaf area, SLA were square-root transformed. Seed mass and leaf lamina density were log-transformed.

Figure S4.


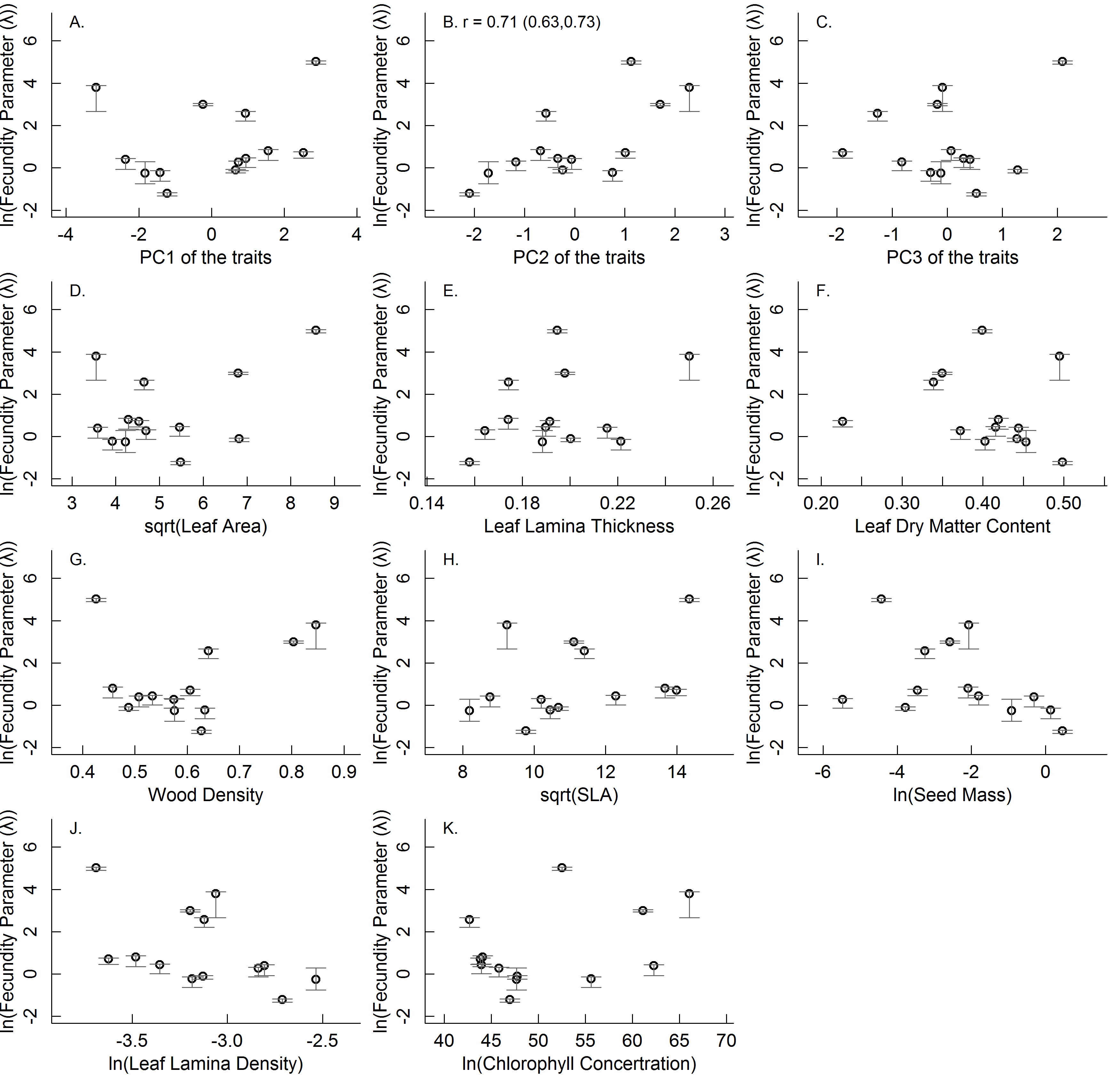


**Fig. S4.** The relationships of the fecundity parameter λ with the first to the third principal components (PC1, PC2, PC3) of the functional traits (A, B, C) and each functional trait (D to K) for tree species in a 20-ha subtropical forest dynamic plot in China. The functional traits included leaf area (D), leaf lamina thickness (E), leaf dry matter content (F), wood density (G), specific leaf area (SLA; H), seed mass (I), leaf lamina density (J) and folia chlorophyll concentration (K). Refer to equation 1 for the fecundity parameter *λ*. Leaf area, SLA were square-root transformed. The fecundity parameter, seed mass and leaf lamina density were log-transformed.

Figure S5.


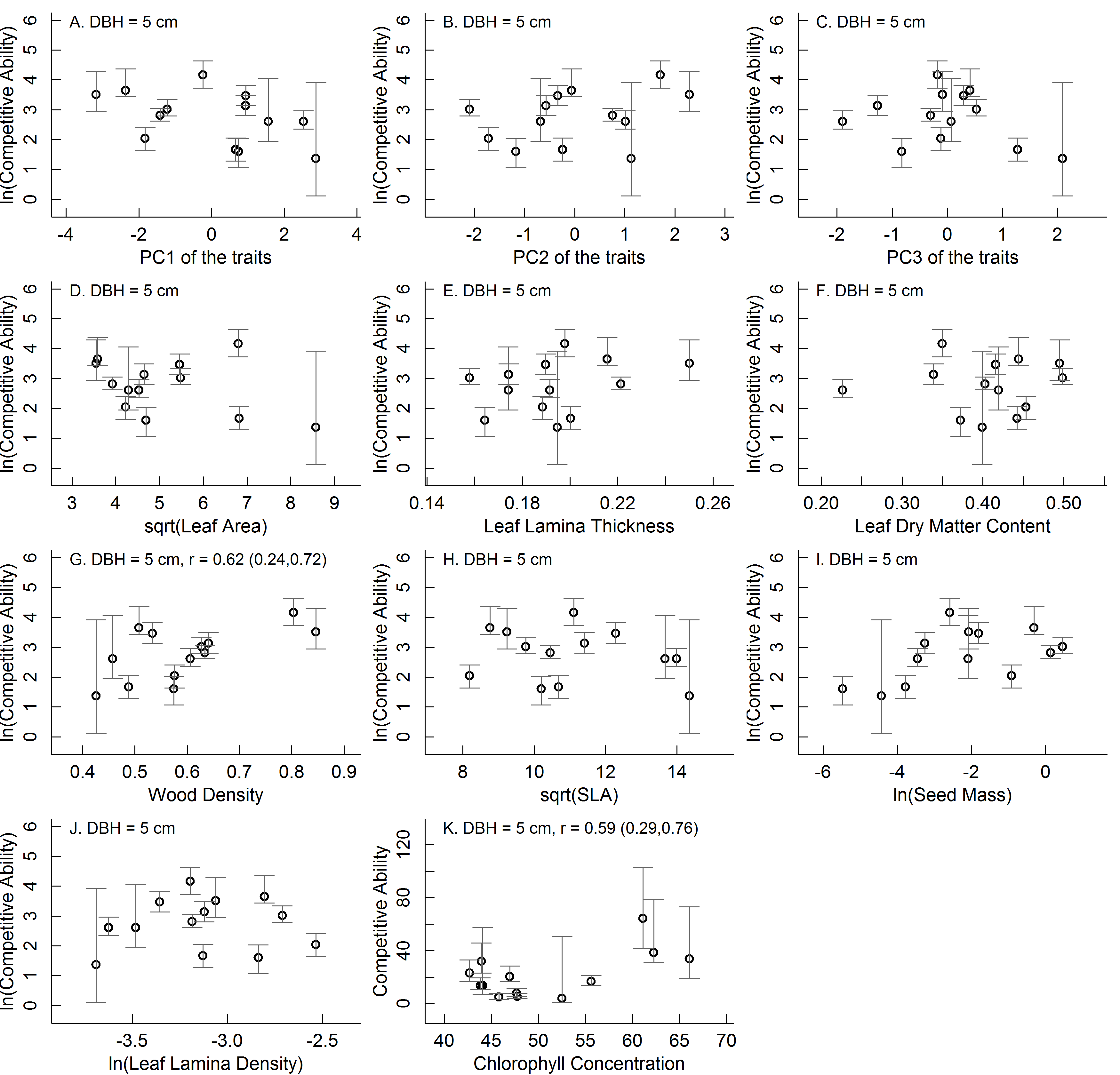


**Fig. S5.** The relationships of the competitive ability when diameter is at 5 cm with the first to the third principal components (PC1, PC2, PC3) of the functional traits (A, B, C) and each functional trait (D to K) for tree species in a 20-ha subtropical forest dynamic plot in China. The functional traits included leaf area (D), leaf lamina thickness (E), leaf dry matter content (F), wood density (G), specific leaf area (SLA; H), seed mass (I), leaf lamina density (J) and folia chlorophyll concentration (K). Competitive ability was the survival odds ratio of an individual with 5 cm diameter at the 97.5^th^ percentile of neighborhood crowding. The competitive ability, leaf area, SLA were square-root transformed. Competitive ability, seed mass and leaf lamina density were log-transformed.

Figure S6.


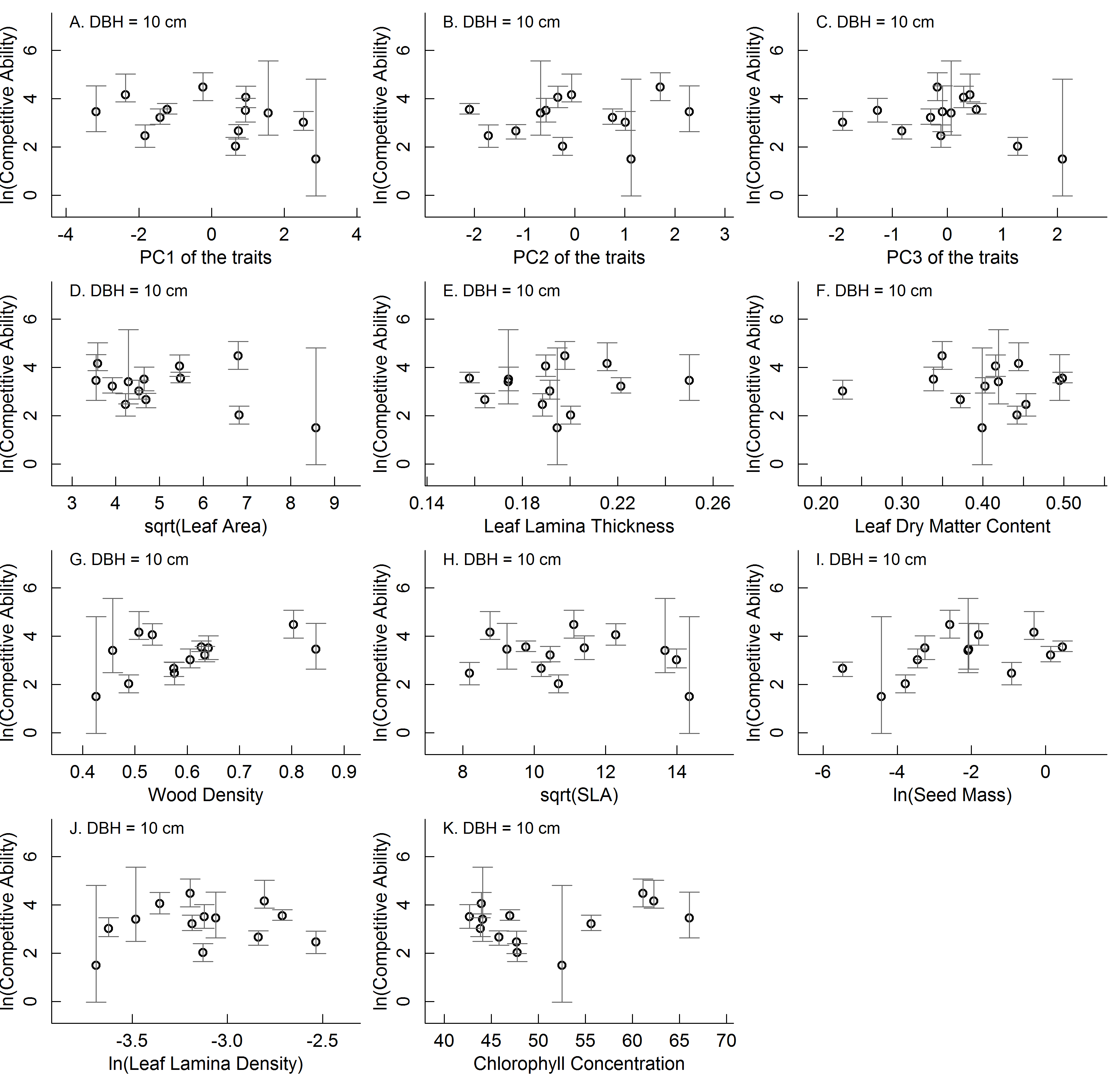


**Fig. S6.** The relationships of the competitive ability when diameter is at 10 cm with the first to the third principal components (PC1, PC2, PC3) of the functional traits (A, B, C) and each functional trait (D to K) for tree species in a 20-ha subtropical forest dynamic plot in China. The functional traits included leaf area (D), leaf lamina thickness (E), leaf dry matter content (F), wood density (G), specific leaf area (SLA; H), seed mass (I), leaf lamina density (J) and folia chlorophyll concentration (K). Competitive ability was the survival odds ratio of an individual with 10 cm diameter at the 97.5^th^ percentile of neighborhood crowding. Competitive ability, leaf area, SLA were square-root transformed. Competitive ability, seed mass and leaf lamina density were log-transformed.

Figure S7.


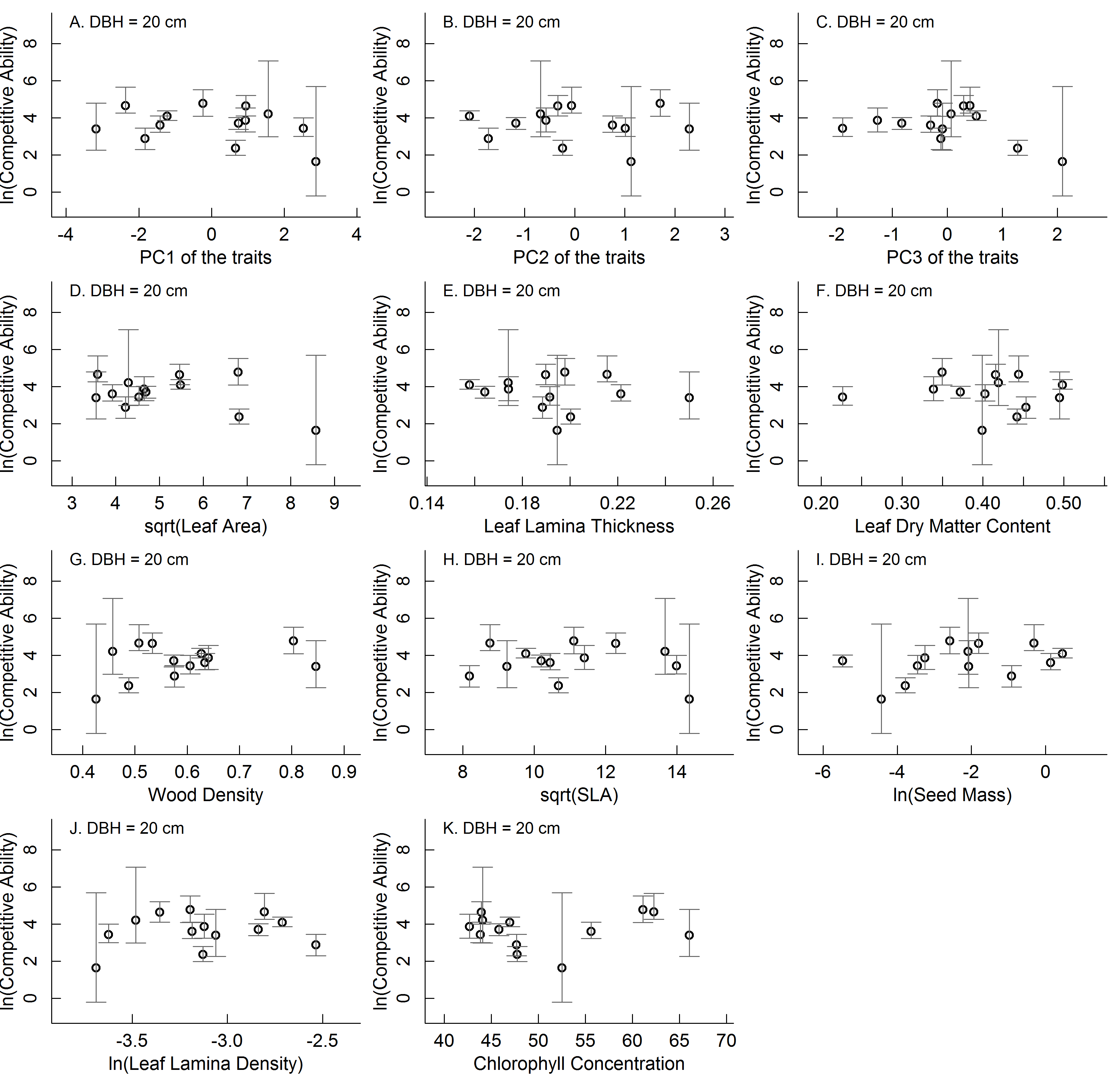


**Fig. S7.** The relationships of the competitive ability when diameter is at 20 cm with the first to the third principal components (PC1, PC2, PC3) of the functional traits (A, B, C) and each functional trait (D to K) for tree species in a 20-ha subtropical forest dynamic plot in China. The functional traits included leaf area (D), leaf lamina thickness (E), leaf dry matter content (F), wood density (G), specific leaf area (SLA; H), seed mass (I), leaf lamina density (J) and folia chlorophyll concentration (K). Competitive ability was the survival odds ratio of an individual with 20 cm diameter at the 97.5^th^ percentile of neighborhood crowding. Leaf area, SLA were square-root transformed. Competitive ability, seed mass and leaf lamina density were log-transformed.

Figure S8


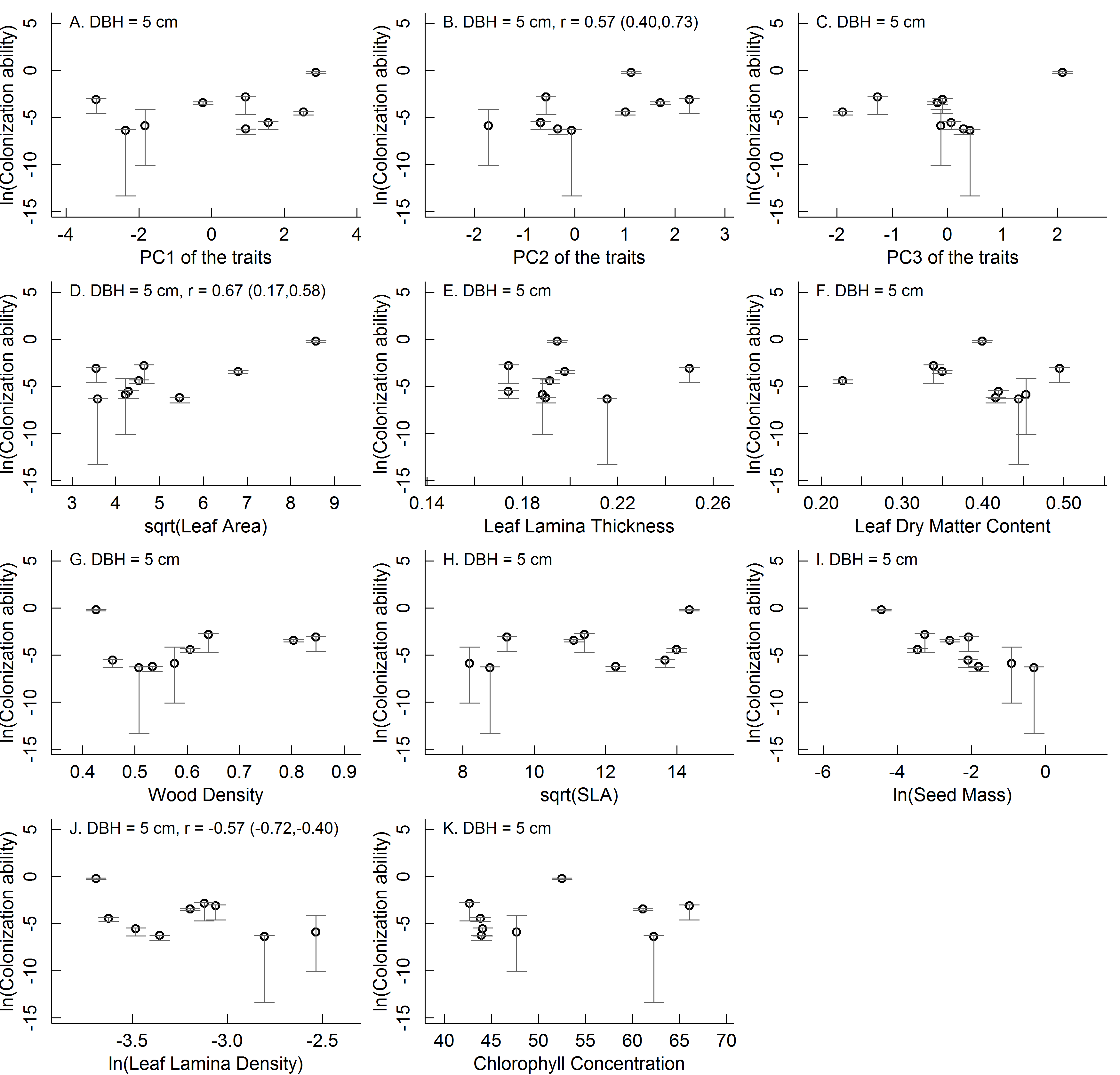


**Fig. S8.** The relationships of the colonization ability when diameter is at 5 cm with the first to the third principal components (PC1, PC2, PC3) of the functional traits (A, B, C) and each functional trait (D to K) for tree species in a 20-ha subtropical forest dynamic plot in China. Colonization ability was expressed as the inverse of the time (*t*) required to colonize a gap. The functional traits included leaf area (D), leaf lamina thickness (E), leaf dry matter content (F), wood density (G), specific leaf area (SLA; H), seed mass (I), leaf lamina density (J) and folia chlorophyll concentration (K). Leaf area, SLA were square-root transformed. Colonization ability, seed mass and leaf lamina density were log-transformed.

Figure S9


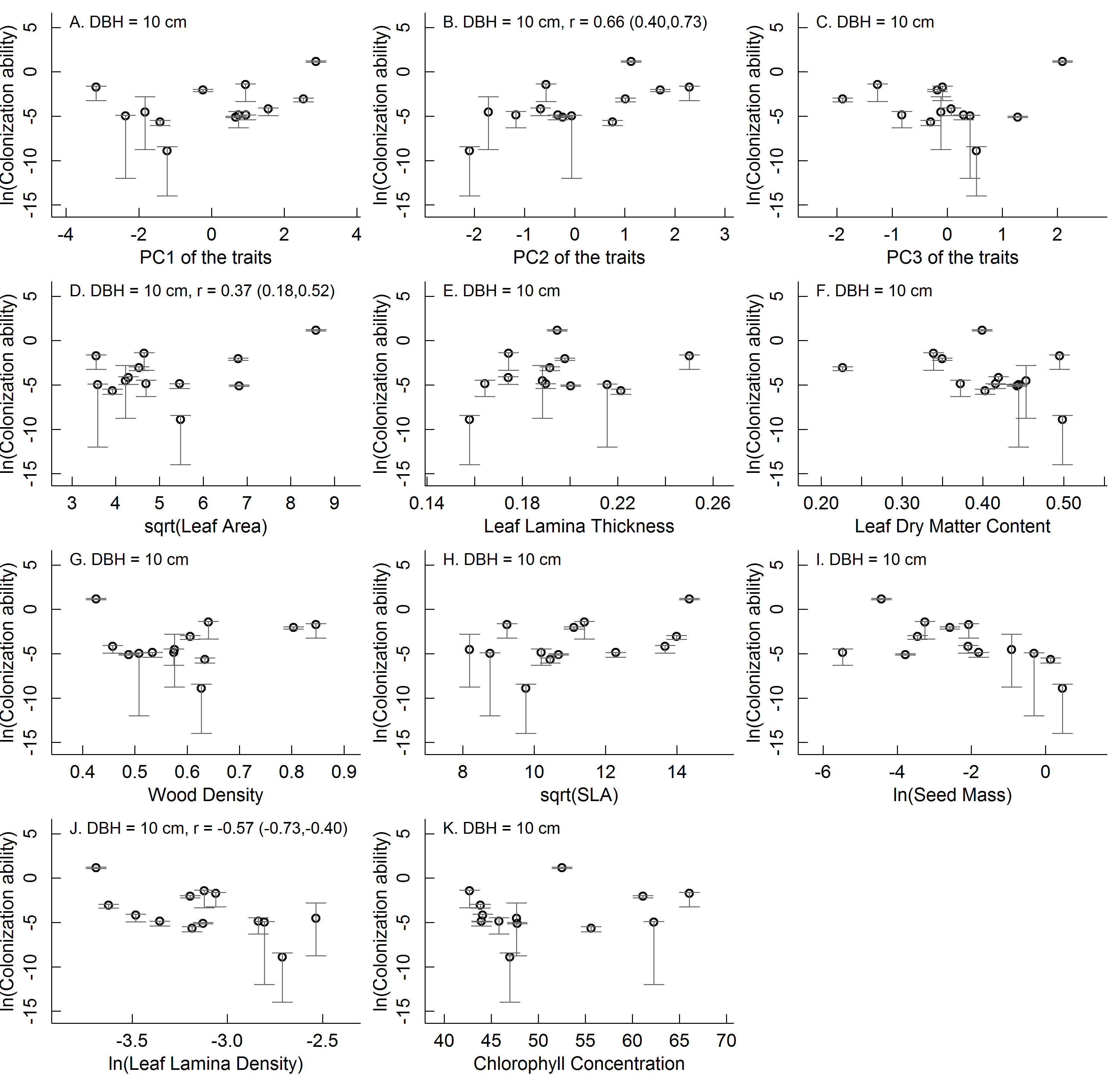


**Fig. S9.** The relationships of the colonization ability when diameter is at 10 cm with the first to the third principal components (PC1, PC2, PC3) of the functional traits (A, B, C) and each functional trait (D to K) for tree species in a 20-ha subtropical forest dynamic plot in China. Colonization ability was expressed as the inverse of the time (*t*) required to colonize a gap. The functional traits included leaf area (D), leaf lamina thickness (E), leaf dry matter content (F), wood density (G), specific leaf area (SLA; H), seed mass (I), leaf lamina density (J) and folia chlorophyll concentration (K). Leaf area, SLA were square-root transformed. Colonization ability, seed mass and leaf lamina density were log-transformed.

Figure S10


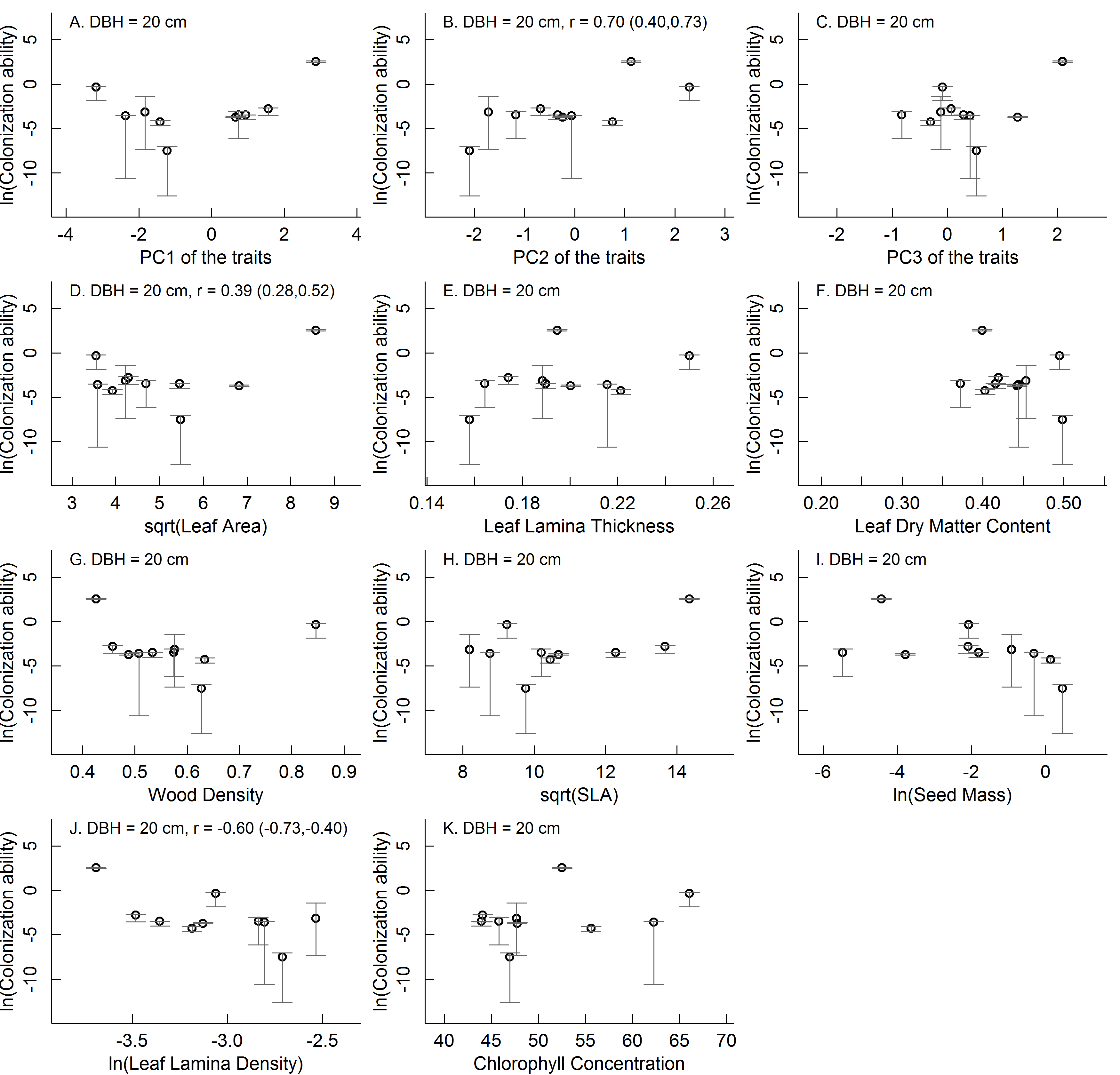


**Fig. S10.** The relationships of the colonization ability when diameter is at 20 cm with the first to the third principal components (PC1, PC2, PC3) of the functional traits (A, B, C) and each functional trait (D to K) for tree species in a 20-ha subtropical forest dynamic plot in China. Colonization ability was expressed as the inverse of the time (*t*) required to colonize a gap. The functional traits included leaf area (D), leaf lamina thickness (E), leaf dry matter content (F), wood density (G), specific leaf area (SLA; H), seed mass (I), leaf lamina density (J) and folia chlorophyll concentration (K). Leaf area, SLA were square-root transformed. Colonization ability, seed mass and leaf lamina density were log-transformed.

Figure S11.


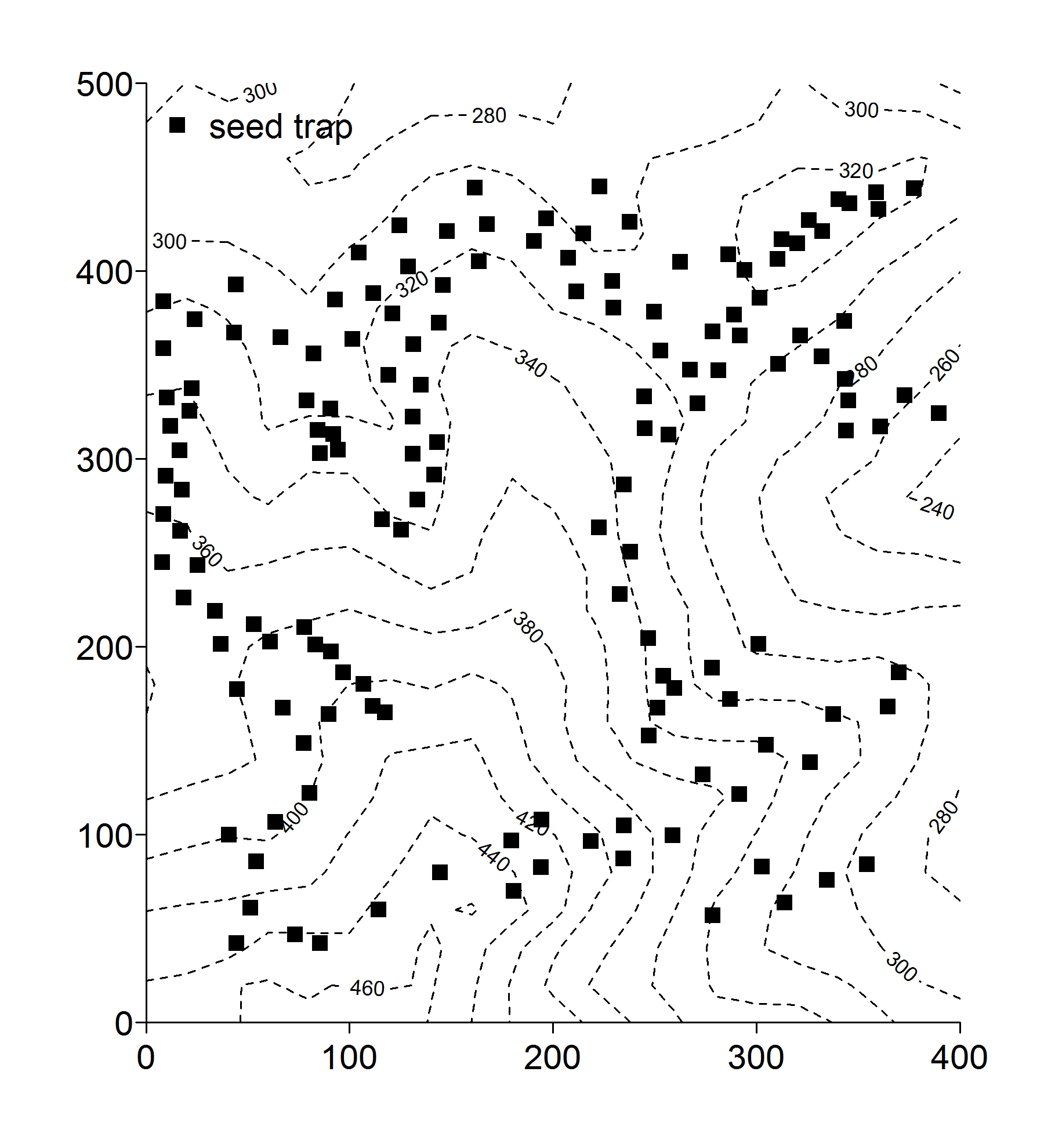


**Fig. S11.** The locations of the 149 seed traps in the Dinghushan 20-ha forest dynamic plot in subtropical China.

**Appendix S1:** Supplemental Methods Section

*Functional trait data*

Only mature seeds and fruits in the traps were counted, since only they can germinate and potentially colonize a site. The number of mature fruits was converted into the number of seeds by multiplying by the average number of seeds per fruit, which was estimated for each of the 13 species based on dissection of 20 fruits per species. Seeds were dried at 80°C for 12 hours and then weighed for seed mass.

Using pole tree pruners or 10 m retractable spear, sun leaves of each species were taken from the six smallest and six largest individuals in the plot, following the leaf trait protocol for the CTFS Barro Colorado Island 50-ha plot (www.ctfs.si.edu). Folia chlorophyll concentration was evaluated as the average chlorophyll concentration measured at three points on each leaf by a portable chlorophyll meter (SPAD 502, Plus Chlorophyll Meter; Konica Minolta, USA)^1^. Leaf lamina thickness, SLA, leaf area, and LDMC were measured on four leaves per tree. For taking wood samples, six individuals haphazardly chosen from outside the plot. For individuals with diameter ≥ 6 cm, cores were drilled from the main stem at 1.3 meters height as wood samples. For individuals with diameter < 6 cm, branches with a diameter of 1 cm and a length of 10 cm were taken as wood samples. The volume of each fresh core was measured using Archimede’s principle, and density calculated after each core was dried for 96 hours and weighed.

*Colonization ability*

We assumed that fecundity was linearly associated with the basal area of a reproductive-sized tree, following many previous studies^2, 3^,

$F_{i}=\lambda B_{i}$ eqn A1

where *F*_i_ is the fecundity of the *i*^th^ tree with basal area *B*_i_, and *λ* is a parameter that scales the number of seeds produced per year to basal area, namely fecundity parameter. We used only trees larger than species-specific reproductive size thresh-holds, which were obtained from experts working in the DHS (Huang, Z. & Cao, H., pers. comm; Table 1). We tested four dispersal kernels widely used for estimating seed dispersal curves: the negative exponential, two dimensional *t* (2Dt), lognormal, and Weibull probability distribution functions^3-5^ (Table A1). In the equations in Table A1, *r_ij_* is the distance between the *i*^th^ reproductive tree and the *j*^th^ seed trap, and *b*_1_ and *b*_2_ are parameters. The expected number of seeds in the *j*^th^ seed trap in one year is

$S_{j}=\sum_{i=1}^{ntrees} F_{i}P_{ij}A_{j}$eqn A2

where *i* indexes trees and *ntrees* is the total number of reproductive trees for a species. *P_ij_* is the probability density for a seed released by the *i*^th^ tree to reach the center of the *j*^th^ seed trap and is calculated from the equations in Table A1. *A*_j_ is the area of the *j*^th^ seed trap, equal to 0.5 m^2^.

*S*_j_ in equation A2 is the theoretical number of seeds for seed trap *j*. However, the observed average number of seeds per year in the *j*^th^ seed trap (*N*_j_) does not precisely equal *S*_j_ but does so with a probability given *S*_j_, which is an expected value^3^. We assumed a Poisson distribution for the probability distribution of *N*_j_ given *S*_j_, and so the likelihood (*L*) of observing the numbers of seeds in these traps was

$L=\prod_{j=1}^{ntraps} \frac{S_{j}^{N_{j}}}{N_{j}!}e^{-S_{j}}$eqn A3.

We maximized *L* using the Nelder-Mead algorithm in the function *optim* in the statistical software, R version 3.0.3^6^ and thus obtained estimates of all parameters including *λ* in equation A1, and *b*_1_ and *b*_2_ for the different dispersal kernels for each species (Table A1).

We used the Akaike Information Criterion (AIC)^7^ to choose the best-supported dispersal kernel (lowest AIC) for each species to be used in subsequent analyses. Seven of the species were best estimated by the 2Dt kernel, four species by the exponential kernel, two species by the lognormal kernel, and one species by the Weibull kernel (Table A2). The fitted models had correlations between observed and predicted seed numbers on log-log scale ranging from 0.38 to 0.65 for each species (Table A2).

We predicted the colonization ability of a species for a single mother tree located at the center of the plot. Because fecundity is influenced by size, three sizes (5, 10, 20 cm as appropriate to each species-specific reproductive size threshold) were used, ultimately producing predicted colonization abilities for these three tree sizes. Fecundity was estimated as the number of seeds produced by an individual of these sizes based on best-fit parameter values for each species and equation A1. A gap of 10×10 m was randomly located in the plot. This gap size was chosen because it approximated the crown projection area of a typical canopy tree in DHS. For calculating the expected number of years needed for successful colonization, we estimated the probability for each seed from a mother tree of a given size to land within the gap (*p*_G_). *p*_G_ is the probability for an area of 10×10m. Numerical integration of the dispersal kernel over the gap area was done with the *adaptIntegrate* function in the R package *cubature*^8^. Even though there is only one mother tree, integration over the gap area (space) is needed in order to obtain the probability for a seed to land in the gap, since we need the cumulative probability of seed arrival for the part of the kernel covering the gap area. Only a single gap was used here because the time needed for at least one seed to colonize a gap was a theoretical expectation that was obtained analytically, not by simulation. Since it was not obtained by simulation, there is no need to model several gaps and take the average.

The arrival of a seed to the gap has the probability *p*_G_ and can be treated as an independent Bernoulli trial. The probability that a seed is the first to arrive in the gap follows a geometric distribution, and the expected number of seeds required for the first arrival is 1/*p*_G_. Hence, the expected number of years for first arrival is

$t=\frac{1}{p_{\text{G}}F}$ eqn A4

where *F* can be calculated by equation A1, and *t* is the expected number of years before the first seed arrives at the gap. We used 1/*t* as a measure of species’ colonization ability. Because fecundity is a function of diameter, 1/*t* is also influenced by tree size, and so we calculated 1/*t* for trees of 5, 10, and 20 cm in diameter. The confidence intervals for these parameters were obtained by the distribution of the parameter estimated for 1000 random resamples of the seed traps.

*Competitive ability*

Competitive ability of a species was estimated as its average survival probability in high crowding conditions. To model survival probability (*s_i_*) as a function of initial diameter and neighborhood crowding index of tree *i*, we used the logistic regression, as follows:

$s_{i}=\frac{\exp(a_{0}+a_{1}\ln(D_{i})+a_{2}\ln(NC_{i}))}{1+\exp(a_{0}+a_{1}\ln(D_{i})+a_{2}\ln(NC_{i}))}$eqn A5

where *D_i_* is the initial diameter of the *i*^th^ individual, respectively; *a*_0_, *a*_1_, and *a*_2_ are parameters. The likelihood of the survival fates of all the individuals of a species alive in the first census is

$L_{ts}=\prod_{i=1}^{ntrees} f_{i}^{s_{i}}(1-f_{i})^{1-s_{i}}$eqn A6

where *f_i_* is the fate of the *i*^th^ individual of a species and equals 1 if the *i*^th^ individual was alive and 0 if it was dead in the second census. Model parameters *a*_0_, *a*_1_, and *a*_2_ were obtained by maximizing equation A6 with the *glm* function in R (Table A3). The confidence intervals for these parameters were also obtained by 1000 resamples of the individuals. The significance of the full model relative to a null model with only the intercept (*a*_0_) was evaluated with a likelihood ratio test using the *lrtest* function in the *lmtest* package. Among these 13 species, the full model had a significantly higher likelihood than the null model for all species except one, *Mallotus paniculatus* (Lam.) Muell. Arg. However, we still used the full model for this species so that all species could be compared within the same model framework.

Competitive ability was estimated from these species-specific fits as the predicted survival probability at the 97.5^th^ percentile of neighborhood crowding (NC) for all individuals across the plot (6088 cm^2^/m) and for trees with diameters of 5, 10, and 20 cm, producing size-specific estimates of survival at high crowding (competitive ability). An NC of 6088 cm^2^/m was an extrapolation for only one species (*M. paniculatus*), which had a maximum NC value of 4533 cm^2^/m. Survival probability of a species at a given diameter was not predicted if that diameter was larger than the observed maximum diameter for that species.

*Data transformation for principal component analysis*

For functional traits, leaf area and SLA were square-root transformed, and seed mass and leaf lamina density were log-transformed to improve normality. For the trade-off, both competitive and colonization abilities were log-transformed. After transformation, all data were standardized by subtracting the mean and dividing by the standard deviation.

Referrences

1. Loh, F. C. W., Grabosky, J. C. & Bassuk, N. L. Using the SPAD 502 meter to assess chlorophyll and nitrogen content of benjamin fig and cottonwood leaves. *Horttechnology* **12**, 682–686 (2002).

2. Ribbens, E., Silander, J. A. & Pacala, S.W. Seedling recruitment in forests: calibrating models to predict patterns of tree seedling dispersion. *Ecology* 75, 1794–1806 (1994).

3. Clark, J. S., Silman, M., Kern, R., Macklin, E. & HilleRisLambers, J. Seed dispersal near and far: patterns across temperate and tropical forests. *Ecology* **80**, 1475–1494 (1999).

4. Clark, J. S., LaDeau, S. & Ibanez, I. Fecundity of trees and the colonization-competition hypothesis. *Ecol. Monogr.* **74**, 415–442 (2004).

5. Muller-Landau, H. C., Wright, S. J., Calderon, O., Condit, R. & Hubbell, S. P. Interspecific variation in primary seed dispersal in a tropical forest. *J. Ecol.* **96**, 653–667 (2008).

6. R Core Team. *R: A Language and Environment for Statistical Computing.* (R Foundation for Statistical Computing, Vienna, Austria, 2014). URL: http://www.R-project.org/.

7. Burnham, K. P. & Anderson, D. R. *Model Selection and Multimodel Inference: A Practical Information-Theoretic Approach*. (Springer, New York, 2002).

8. Narasimhan, B. *cubature: Adaptive multivariate integration over hypercubes.* R package version 1.1-2 (2013).URL:http://CRAN.R-project.org/package=cubature.

**Table A1.** The functional forms of the probability distribution functions used as seed dispersal kernels for tree species in a Chinese subtropical forest. *r*_ij_ is the distance between the *i*^th^ reproductive tree and the *j*^th^ seed trap, and *b*_1_ and *b*_2_ are parameters.

| Probability distribution | Distribution function |
| --- | --- |
| Exponential | $P_{ij}=\frac{1}{2\pi b_{1}r_{ij}}e^{-\frac{r_{ij}}{b_{1}}}$ |
| Two dimensional *t* | $P_{ij}=\frac{b_{2}}{\pi b_{1}(1+\frac{r_{ij}^{2}}{b_{1}})^{b_{2}+1}}$ |
| Lognormal | $P_{ij}=\frac{1}{(2\pi)^{3/2}r_{ij}^{2}}\frac{1}{b_{2}}\exp(-\frac{(\ln r_{ij}-b_{1})^{2}}{2b_{2}^{2}})$ |
| Weibull | $P_{ij}=\frac{1}{2\pi r_{ij}}\frac{b_{2}}{b_{1}}(\frac{r_{ij}}{b_{1}})^{b_{2}-1}\exp(-(\frac{x}{b_{1}})^{b_{2}})$ |

**Table A2.** Parameter values for the best-fitting dispersal kernels and survival models for the 13 focal tree species in a Chinese subtropical forest. Species abbreviations are in Table 1. For the dispersal models (Table A1): *λ* is the fecundity parameter in equation A1; LNM: lognormal; EXP: exponential; 2Dt: two dimensional t; WBL: Weibull; *k* is the parameter of negative binomial distribution; *b*_1_ and *b*_2_: the dispersal parameters for the corresponding dispersal kernels; *r*: the Pearson’s correlation coefficient between the observed and the fitted numbers of seeds on log-log scale, and all correlation coefficients were statistically significant (*p*< 0.05). Latin binomials for the species were in Table 1 in the main text.

| Species | Kernel | *λ* | *b*_1_ | *b*_2_ | *r* |
| --- | --- | --- | --- | --- | --- |
| MP | EXP | 151.52  (133.88-156.05) | 227.55  (1.90-242.7) | NA | 0.58 |
| ML | 2Dt | 44.43  (14.40-60.61) | 0.21  (0-0.63) | 0.08  (0.03-0.30) | 0.48 |
| OG | 2Dt | 1.55  (1.02-1.60) | 7.02  (3.90-9.84) | 0.21  (0.17-0.31) | 0.60 |
| AC | 2Dt | 19.97  (18.96-20.79) | 21.8  (10.56-34.68) | 0.1  (0.08-0.12) | 0.52 |
| SS | LNM | 1.32(0.88-1.38) | 7.27  (5.42-8.39) | 3.43  (2.22-5.14) | 0.40 |
| CC | 2Dt | 1.48  (0.93-1.56) | 16.36  (14.98-28.67) | 0.29  (0.30-0.45) | 0.58 |
| MC | LNM | 0.78  (0.47-1.34) | 3.9  (3.00-7.88) | 1.35  (0.03-4.00) | 0.52 |
| ER | 2Dt | 0.90  (0.78-0.92) | 24.47  (20.37-43.62) | 0.11  (0.11-0.16) | 0.65 |
| AQ | EXP | 13.07  (9.15-12.80) | 74.2  (31.33-84.31) | NA | 0.40 |
| AA | 2Dt | 0.80  (0.53-0.80) | 2.88  (1.76-22.90) | 0.06  (0.05-0.09) | 0.38 |
| AS | 2Dt | 2.22  (1.42-2.37) | 27.48  (6.5-89.51) | 0.18  (0.14-0.46) | 0.52 |
| AY | EXP | 2.04  (1.58-2.15) | 131.71  (80.19-145.19) | NA | 0.40 |
| Cc | 2Dt | 0.30  (0.26-0.31) | 6409.94  (5061-6759.46) | 4.92  (4.58-5.87) | 0.47 |

Table A3. The estimates and their confidence intervals for the parameters in the survival model, *a_0_*, *a_1_* and *a_2_* are the parameters for equation A6. The numbers in the parentheses below the parameter values were the 95% confidence intervals obtained from 1000 bootstraps. Species latin binomials were in Table A1 in the main text.

| Species | *a_0_* | *a_1_* | *a_2_* |
| --- | --- | --- | --- |
| MP | -0.32  (-12.76-9.04) | 0.20  (-0.38-1.28) | 0.16  (-0.99-1.69) |
| ML | -5.32  (-11.71-0.91) | -0.08  (-0.56-0.42) | 1.03  (0.30-1.80) |
| OG | -4.86  (-8.22--1.11) | 0.85  (0.68-1.04) | 0.80  (0.35-1.21) |
| AC | -9.03  (-12.64--4.71) | 0.44  (0.24-0.67) | 1.43  (0.91-1.88) |
| SS | -3.62  (-5.54--0.74) | 1.53  (1.18-1.99) | 0.32  (0.02-0.43) |
| CC | -1.83  (-10.37--0.40) | 0.73  (0.53-0.99) | 0.50  (0.34-1.52) |
| MC | -0.57  (-2.05-3.52) | 0.61  (0.35-0.87) | 0.19  (-0.30-0.35) |
| ER | 1.05  (0.10-3.19) | 0.51  (0.31-0.71) | -0.02  (-0.27-0.07) |
| AQ | -5.99  (-8.76--3.46) | 0.53  (0.29-0.78) | 0.95  (0.64-1.28) |
| AA | 0.07  (-0.94-1.30) | 0.58  (0.36-0.81) | 0.21  (0.08-0.32) |
| AS | -1.03  (-10.14-7.78) | 1.15  (0.65-2.25) | 0.20  (-0.84-1.28) |
| AY | -5.90  (-8.67--3.66) | 0.59  (0.42-0.78) | 0.87  (0.60-1.20) |
| Cc | -1.73  (-2.47--0.78) | 0.77  (0.52-0.98) | 0.40  (0.36-0.45) |
